# Reprograming of Tumor-Associated Macrophages in Breast Tumor-Bearing Mice under Chemotherapy by Targeting Heme Oxygenase-1

**DOI:** 10.3390/antiox10030470

**Published:** 2021-03-16

**Authors:** Seung Hyeon Kim, Su-Jung Kim, Jeongmin Park, Yeonsoo Joe, So Eui Lee, Soma Saeidi, Xiancai Zhong, Seong Hoon Kim, Sin-Aye Park, Hye-Kyung Na, Hun Taeg Chung, Young-Joon Surh

**Affiliations:** 1 Cancer Research Institute, Seoul National University, Seoul 03087, Korea; shjbeh@naver.com; 2 Research Institute of Pharmaceutical Sciences, College of Pharmacy, Seoul National University, Seoul 08826, Korea; nynna79@snu.ac.kr (S.-J.K.); thdml0429@naver.com (S.E.L.); saeidi@snu.ac.kr (S.S.); zhongxiancai@163.com (X.Z.); daduhoon@snu.ac.kr (S.H.K.); 3 Department of Biological Sciences, University of Ulsan, Ulsan 44610, Korea; pjm2368@mail.ulsan.ac.kr (J.P.); jcantibody@ulsan.ac.kr (Y.J.); chung@ulsan.ac.kr (H.T.C.); 4 Department of Molecular Medicine and Biopharmaceutical Sciences, Graduate School of Convergence Science and Technology, Seoul National University, Seoul 08826, Korea; 5 Department of Biomedical Laboratory Science, College of Medical Sciences, Soonchunhyang University, Asan 31538, Korea; sappark@sch.ac.kr; 6 Department of Food Science and Biotechnology, College of Knowledge-Based Services Engineering, Sungshin Women’s University, Seoul 01133, Korea; nhkdec28@gmail.com

**Keywords:** breast cancer, chemotherapy, tumor-associated macrophages, phagocytosis, tumor cell debris, heme oxygenase-1

## Abstract

Tumor-associated macrophages (TAMs) represent one of the most abundant components of the tumor microenvironment and play important roles in tumor development and progression. TAMs display plasticity and functional heterogeneity as reflected by distinct phenotypic subsets. TAMs with an M1 phenotype have proinflammatory and anti-tumoral properties whereas M2-like TAMs exert anti-inflammatory and pro-tumoral functions. Tumor cell debris generated during chemotherapy can stimulate primary tumor growth and recurrence. According to our previous study, phagocytic engulfment of breast tumor cell debris by TAMs attenuated chemotherapeutic efficacy through the upregulation of heme oxygenase-1 (HO-1). To verify the impact of HO-1 upregulation on the profile of macrophage polarization during cytotoxic therapy, we utilized a syngeneic murine breast cancer (4T1) model in which tumor bearing mice were treated with paclitaxel (PTX). PTX treatment markedly downregulated the surface expression of the M1 marker CD86 in infiltrated TAMs. Notably, there were significantly more cytotoxic CD8^+^ T cells in tumors of mice treated with PTX plus the HO-1 inhibitor, zinc protophorphyrin IX (ZnPP) than in mice treated with PTX alone. Interestingly, the tumor-inhibiting efficacy of PTX and ZnPP co-treatment was abrogated when macrophages were depleted by clodronate liposomes. Macrophage depletion also decreased the intratumoral CD8^+^ T cell population and downregulated the expression of *Cxcl9* and *Cxcl10.* The expression of the M1 phenotype marker, CD86 was higher in mice injected with PTX plus ZnPP than that in mice treated with PTX alone. Conversely, the PTX-induced upregulation of the M2 marker gene, *Il10* in CD11b^+^ myeloid cells from 4T1 tumor-bearing mice treated was dramatically reduced by the administration of the HO-1 inhibitor. Genetic ablation of HO-1 abolished the inhibitory effect of 4T1 tumor cell debris on expression of M1 marker genes, *Tnf* and *Il12b*, in LPS-stimulated BMDMs. HO-1-deficient BMDMs exposed to tumor cell debris also exhibited a diminished expression of the M2 macrophage marker, CD206. These findings, taken all together, provide strong evidence that HO-1 plays a pivotal role in the transition of tumor-inhibiting M1-like TAMs to tumor-promoting M2-like ones during chemotherapy.

## 1. Introduction

The tumor microenvironment (TME) is a complex niche that is comprised of different types of stromal cells and their supportive matrix as well as cancer cells [1,2]. Both cancer cells and surrounding non-tumorigenic cells crosstalk by secreting soluble factors including chemokines and cytokines as well as growth factors [3]. Such interactions often confer a survival advantage for cancer cells and resistance to anticancer therapy. Macrophages recruited into TME, termed tumor-associated macrophages (TAMs), represent the major innate immune component of the stroma and play important roles in tumor development and progression as well as intratumoral immune status [4,5,6]. There has been a growing body of evidence supporting that high density of TAMs is associated with poor prognosis, drug resistance, enhanced angiogenesis, and metastasis in cancer [7,8].

TAMs exhibit pronounced plasticity and dynamic nature. Further, TAMs have functional heterogeneity which is reflected by distinct phenotypic subsets. In response to different stimuli, macrophages are polarized into two subtypes [9,10]. TAMs with an M1 phenotype are known as classically activated macrophages capable of phagocytosing pathogens and have proinflammatory and anti-tumoral properties. In contrast, M2-like (or alternatively activated) TAMs have anti-inflammatory properties and exert pro-tumoral and tumor supporting functions. Depending on the microenvironment, macrophages can reprogram their phenotype toward the proinflammatory M1 phenotype or the anti-inflammatory M2 phenotype [10]. Macrophage reprogramming is a highly coordinated process mediated by a subset of orchestrated signaling molecules [10,11,12]. Cancer-associated fibroblasts, one of the most abundant cell types in the tumor stroma, play key roles in the macrophage reprogramming to instruct the immunosuppressive TME [13]. The polarized macrophages also direct Th1/Th2 polarization which regulates the adaptive immune responses [14].

TAMs in the vicinity of progressive tumors usually acquire an M2 phenotype, which enables them to perform immunosuppressive and tumor-promoting functions [15,16]. Once recruited by tumor cells secreting chemoattractants, TAMs are educated in TME. Thus, tumor cells can reprogram proinflammatory M1-like macrophages into anti-inflammatory M2-like macrophages, provoking local immune suppression [17,18]. Phenotypic switching of pro-tumoral TAMs to the proinflammatory and tumor inhibiting M1 phenotype, thereby restoring immune surveillance and tumoricidal effects, has been suggested as a promising therapeutic approach towards tumor regression [16,17].

Chemotherapy is one of the common therapeutic options practiced for the treatment of malignancies. However, the therapeutic sensitivity of tumors to chemotherapy is often diminished by disruption of patient’s anti-tumor immunity. This is mainly due to the complex interaction of cancer cells with different immune components of the TME, particularly TAMs [19,20]. Though chemotherapy is supposed to kill tumor cells, the resulting dead/dying tumor cells or debris, the inevitable byproduct may paradoxically stimulate tumor growth and recurrence by triggering release of inflammatory cytokines by macrophages [21,22]. Osteopontin was found to be a critical mediator of debris-stimulated tumor growth [22]. Dying/dead tumor cells are thought to promote the proliferation and survival of the residual living tumor cells and thus preserve them as the seeds for tumor repopulation [23]. They may establish an immunosuppressive TME which is favorable for the survival of living tumor cells, and trigger the activation of dormant tumor or tumor-initiating (stem-like) cells [23]. As chemotherapy develops an immunosuppressive TME, reprogramming of TAMs to acquire an immunocompetent phenotype is a promising strategy to potentiate therapeutic efficacy.

Heme oxygenase-1 (HO-1) is the rate-limiting enzyme that catalyzes the breakdown of free heme into carbon monoxide, ferrous iron, and biliverdin. HO-1 has cytoprotective, anti-apoptotic, anti-oxidative, and anti-inflammatory functions in physiological conditions. However, HO-1 also plays an opposite role in the pathogenesis and progression of several types of malignancies and resistance to chemotherapy [24]. For instance, lung cancer cells expressing high levels of HO-1 were less sensitive to cisplatin, and the genetic or pharmacologic inhibition of HO-1 activity by siRNA or zinc protoporphyrin IX (ZnPP), respectively augmented the cytotoxic effect of this anticancer drug. Administration of the HO-1 inhibitor ZnPP significantly suppressed the growth of tumors in a murine xenograft lung cancer model [discussed in 24 and reference therein].

We previously reported that phagocytic engulfment of tumor cell debris by macrophages hampered M1-like polarization which was associated with HO-1 upregulation [25]. This may diminish the chemotherapeutic efficacy. In the present study, we attempted to verify these findings in vivo by use of the HO-1 inhibitor, ZnPP and by the depletion of macrophages.

## 2. Materials and Methods

### 2.1. Bioinformatic Analysis

Microarray data were extracted from the Gene Expression Omnibus database (GSE 43816; National Center for Biotechnology Information, Bethesda, MD, USA; https://www.ncbi.nlm.nih.gov/.geo/ (accessed on 30 April 2019)). Heatmaps were generated using the R software (Bioconductor, ComplexHeatmap package).

### 2.2. Kaplan–Meier (Relapse-Free Survival) Analysis

Kaplan–Meier analysis (http://kmplot.com/analysis (accessed on 29 January 2021)) was performed based on the RFS (relapse-free survival) data of patients with low and high *HMOX1* mRNA (HMOX1 probe set 203665_at) expression.

### 2.3. Syngeneic Murine Breast Tumor Model

BALB/c mice (6~9 weeks old) were purchased from Orient Bio (Gyeonggi-Do, Sungnam city, South Korea). HO-1 KO mice, in which the HO-1 gene was deleted by targeted gene knockout, were kindly provided by Dr. M.A. Perrella (Harvard Medical School, Boston, MA, USA). 4T1 murine mammary adenocarcinoma cells (1 × 10^5^) were implanted into the mammary glands of female BALB/c mice. The tumors were grown for 10 days before mice were treated with chemotherapeutic agents. Tumor size was measured by caliper (width × length × height × 0.52 = mm^3^). All the animals were maintained according to the relevant institutional animal care guidelines. Animal experimental procedures were approved by the institutional animal care and use committee at Seoul National University (IACUC number: SNU-170710-2).

### 2.4. Macrophages Depletion

Established 4T1 tumors were injected with clodronate liposomes (1 mg/mouse; FormuMax Scientific, Inc., Sunnyvale, CA, USA) 3 times at 4-day intervals following the first intraperitoneal administration of paclitaxel (PTX; 5 mg/kg). ZnPP (40 mg/kg) was injected intraperitoneally on daily basis throughout the experiment. Depletion of macrophages was confirmed by flow cytometry.

### 2.5. Cell Culture

To culture primary bone marrow derived macrophages (BMDMs), we obtained bone marrow cells by flushing the long bones of mice (6~12 weeks old). Bone marrow cells were plated in DMEM supplemented with 10% fetal bovine serum (FBS), 100 μg/mL streptomycin, 100 U/mL penicillin and 20 ng/mL M-CSF (Biolegend, San Diego, CA, USA) and cultured for 7 days to allow for macrophage differentiation. Murine 4T1 cells were purchased from the American Type Culture Collection (ATCC, Manassas, VA, USA) and cultured in DMEM supplemented with 10% FBS, 100 μg/mL streptomycin and 100 U/mL penicillin. Cells were maintained at 37 °C in a humidified atmosphere of 5% CO_2._

### 2.6. Macrophage Polarization

To generate M1-polarized macrophages, BMDMs were treated with LPS (100 ng/mL) for 24 h as described previously [25].

### 2.7. Generation of Tumor Cell Debris

Tumor cell debris was generated by incubating murine 4T1 breast cancer cells (1 × 10^7^) in medium supplemented with PTX (1 mM) for 24 h. The presence of dead cells among the debris was analyzed by the Annexin V/propidium iodide (PI) assay.

### 2.8. Isolation of Single Cells from Mouse Tumors

Tumors isolated from mice were dissociated into single cells as described previously [21]. Specifically, tumor implanted mice were euthanized by CO_2_ inhalation. Collected tumors were minced, mechanically disaggregated, and passed through a 40 µm filter using DMEM supplemented with 2% FBS to prepare a single cell suspension.

### 2.9. Flow Cytometric Analysis

The cells were washed with phosphate-buffered saline (PBS) containing 1% FBS. The CD16/32 Fc receptor was blocked by anti-mouse CD16/32 antibody (Biolegend; San Diego, CA, USA). The cells were then stained for 30 min incubation with the following fluorescence-conjugated antibodies: anti-mouse CD45 APC, anti-mouse CD45.2 BV510, anti-mouse CD11b Alexa Fluor700, anti-mouse F4/80 PerCP/Cy5.5, anti-mouse F4/80 APC, anti-mouse Ly-6C PE/Cy7, anti-mouse Ly-6G APC/Cy7, anti-mouse CD3ε Alexa Fluor 700, anti-mouse CD8a PE/Cy7, anti-mouse CD4 PE, anti-mouse CD206 FITC and anti-mouse CD36 PE. After washing with PBS, cells were analyzed by flow cytometry. Dead cells were excluded by DAPI (Thermo Fisher Scientific; Waltham, MA, USA) staining.

For intracellular staining, the cells were incubated with ionomycin (1 μg/mL) and phorbol myristate acetate (PMA; 100 ng/mL) for 4 h at 37 °C in a humidified atmosphere of 5% CO_2_ in the presence of a protein transport inhibitor cocktail (Brefeldin A and Monensin). Thermo Fisher Scientific, Waltham, MA, USA). The cells were fixed and permeabilized with a fixation/permeabilization buffer set (Thermo Fisher Scientific; Waltham, MA, USA), and then stained with the following antibodies: anti-mouse IL-12p40 PE, anti-mouse TNF-α FITC, anti-mouse IFN-γ APC (all from Biolegend, San Diego, CA, USA) and anti-mouse Foxp3 FITC-conjugated antibody (Thermo Fisher Scientific; Waltham, MA, USA).

To stain intracellular HO-1, a fixation/permeabilization buffer set (Thermo Fisher Scientific, Waltham, MA, USA) was used according to manufacturer’s instructions. Briefly, the cells were stained with anti-HO-1 antibody in buffer, washed with PBS and analyzed by flow cytometry. FACS Calibur, LSR Fortessa X-20 and FACS Aria III (BD; Franklin Lakes, NJ, USA) was used for the aforementioned analyses. All samples were quantified using the FlowJo^TM^ software package (Tree Star; Ashland, OR, USA).

### 2.10. Cell Sorting

Single cells obtained from tumors were stained with DAPI, anti-CD45 APC and anti-CD11b Alexa Fluor700 and subjected to FACS using a FACS Aria III (BD; Franklin Lakes, NJ, USA). Live cells were identified as CD45^+^ CD11b^+^.

### 2.11. Immunofluorescence Analysis

For immunofluorescence staining, paraffin-embedded tumor tissue sections were boiled in 10 mM sodium citrate buffer (pH 6), subjected to serial washing, blocked with 5% FBS in PBST (PBS + 0.1% Tween 20) and stained with CD8 Alexa Fluor 594 (Biolegend; San Diego, CA, USA) and DAPI (Thermo Fisher Scientific; Waltham, MA, USA) overnight at 4 °C. Immunofluorescence images were analyzed by a Zeiss LSM 710 confocal microscope.

### 2.12. Phagocytosis Assay

PTX-induced tumor cell debris was stained with 1 μL of 1 mg/mL pHrodo-SE (Thermo Fisher Scientific, Waltham, MA, USA) for 30 min at room temperature. BMDMs were co-cultured with pHrodo-SE-labeled tumor cell debris. After incubation for 8 h, the phagocytic activity of BMDMs was assessed by flow cytometry or confocal microscopy.

### 2.13. Quantitative PCR

The total RNA isolated from cells and tumor tissues was reverse transcribed using murine leukemia virus reverse transcriptase (Promega; Madison, WI, USA). qPCR analysis was conducted by a 7300 Real-Time PCR System (Thermo Fisher Scientific, Waltham, MA, USA). Gene expression was normalized relative to internal control. The sequences of primers used are as Table 1.

### 2.14. Statistical Analysis

The statistical significance was determined by one-way ANOVA analysis with a Tukey’s post hoc test. The Student’s *t*-test was used to compare data from control and experimental group. The analyses were conducted by SigmaPlot 12 (Systat Software; San Jose, CA, USA). Data are presented as the mean ± SEM (standard error of the mean).

## 3. Results

### 3.1. Chemotherapy Induces TAM Polarization toward the M2 Phenotype

**Figure 1:**
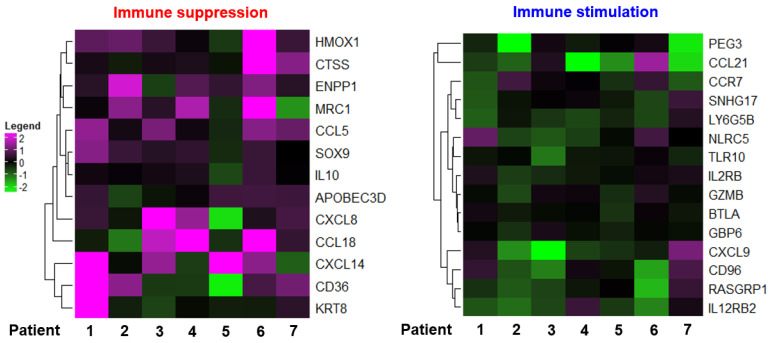
Heatmap of genes from breast cancer patients who underwent chemotherapeutic treatments (GSE 43816).

To investigate the impact of chemotherapy on host immunity, we analyzed a public gene expression dataset obtained from breast cancer patients who received chemotherapy (GSE 43816). We found that the expression levels of immunosuppressive genes were largely elevated, while some of the genes associated with immune stimulation were downregulated after chemotherapy (Figure 1). To verify the impact of cytotoxic therapy on anti-tumor immunity in the TME, we utilized a syngeneic murine breast cancer (4T1) model in which mice were treated with PTX (Figure S1A), one of the most commonly used chemotherapeutic agents. As expected, systemic administration of PTX inhibited the growth of 4T1 tumors (Figure S1B). To determine whether PTX treatment could alter the proportion of TAMs in a 4T1 breast cancer model, we stained infiltrating TAMs with fluorochrome-conjugated antibodies for flow cytometric analysis. We found that administration of PTX downregulated the surface expression of the M1 marker CD86 in infiltrated TAMs (Figure S1C).

### 3.2. Phagocytic Engulfment of Tumor Cell Debris Re-Educates Macrophages toward M2-Like Polarization

**Figure 2:**
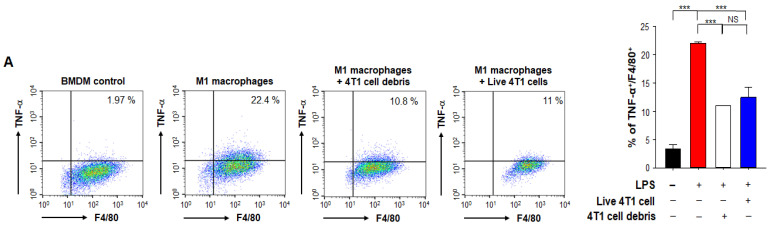

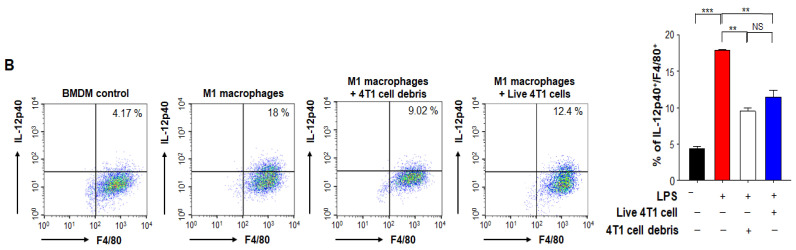
Effects of PTX-generated breast tumor cell debris on macrophage polarization. Murine BMDMs and live 4T1 breast cancer cells or those killed by PTX treatment were co-cultured for 24 h. After co-culture, **t**he proportions of macrophages expressing TNF-α (**A**) and IL-12p40 (**B**) were analyzed by flow cytometry. **, *** Significantly different between groups compared (** *p* < 0.01; *** *p* < 0.001). NS; not significant.

TAMs undergo polarization into an M2 phenotype that suppresses anti-tumor immunity. We hypothesized that phagocytic engulfment of tumor cell debris by macrophages in TME may facilitate such polarization. To test this hypothesis, we co-cultured murine BMDMs and 4T1 breast cancer cells killed by PTX treatment for 24 h. PTX-induced cell death was confirmed by flow cytometric analysis of annexin V/PI-stained cells (Figure S2A). After co-culture, we examined the markers of M1- and M2-polarized macrophages. Tumor necrosis factor alpha (TNF-α) and interleukin 12 (IL-12) are two major macrophage-derived mediators of inflammatory responses in mammals [26]. We observed significant decreases in the proportions of BMDMs expressing TNF-α (Figure 2A) and IL-12p40 (Figure 2B). Proteins secreted from tumor cells have been shown to inhibit M1 polarization to reduce the anti-tumor immune response [27], suggesting that TME-mediated M1 TAM inactivation is accelerated by tumor cell debris produced during chemotherapy. We also confirmed that the proportion of macrophages expressing TNF-α (Figure 2A) or IL-12p40 (Figure 2B) following co-culture with viable 4T1 breast cancer cells was decreased.

**Figure 3:**
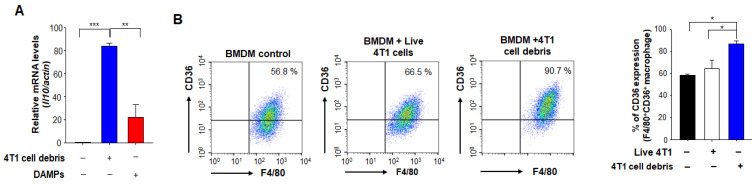
Effects of PTX-generated tumor cell debris on expression of CD36 in BMDMs. (**A**) The *Il10* mRNA levels of BMDMs incubated for 8 h with or without 4T1 cell debris or DAMPs from 4T1 tumor cell debris were analyzed by qPCR. (**B**) The expression of CD36 on macrophages co-cultured with viable 4T1 cells or 4T1 tumor cell debris for 24 h was analyzed by flow cytometry. *, **, *** Significantly different between groups compared (* *p* < 0.05; ** *p* < 0.01; *** *p* < 0.001).

In another experiment, BMDMs co-incubated with breast tumor cell debris exhibited a robust upregulation of *Il10* which is known to stimulate the M2 polarization (Figure 3A). Danger-associated molecular patterns (DAMPs) released from dead cells contribute to promoting tumors by triggering immunosuppression [28]. Macrophages treated with DAMPs also induced *Il10* mRNA expression (Figure 3A). These results suggest that engulfment of tumor cell debris by TAMs may provoke an immunosuppressive microenvironment through M2 polarization whereas it suppresses the M1 polarization. To verify the expression of phosphatidylserine (PS) recognition receptor on the surface of TAMs, we measured the expression of CD36 in BMDMs treated with tumor debris. The expression levels of CD36 were higher in macrophages exposed to breast cancer cell debris compared to those co-cultured with viable breast cancer cells (Figure 3B).

### 3.3. Chemotherapy Enhances Expression of HO-1 Which Is Associated with Poor Prognosis

**Figure 4:**
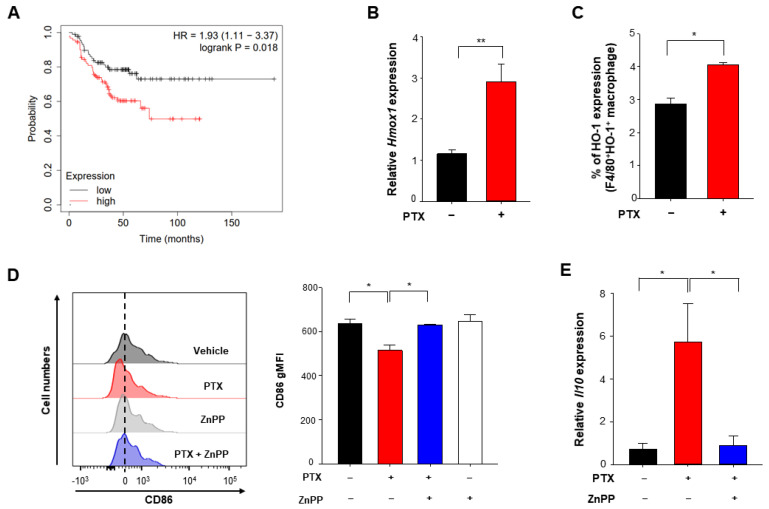
Induction of HO-1 by chemotherapy and its effects on macrophage polarization. (**A**) Kaplan–Meier analysis (http://kmplot.com/analysis (accessed on 29 January 2021)) of RFS (relapse-free survival) based on low or high *HMOX1* mRNA (HMOX1 probe set 203665_at) expression in breast cancer patients who had chemotherapy. For animal experiments (**B**–**E**), 4T1 murine cancer cells were implanted into the mammary glands of female mice. Mice implanted with 4T1 cancer cells received vehicle or PTX (5 mg/kg, *i.p.*) for 5 days with or without daily intraperitoneal injection of ZnPP (40 mg/kg). Mice were sacrificed on day 15 after PTX injection, and breast tumor tissues were analyzed. (**B**) Relative mRNA levels of *Hmox1* in CD45^+^CD11b^+^ tumor-associated myeloid cells isolated from 4T1 tumors were assessed by qPCR. (**C**) The HO-1 expression levels in TAMs from 4T1 tumor-bearing mice injected with or without PTX were analyzed by flow cytometry. (**D**) CD45^+^CD11b^+^Ly6G^−^Ly6C^−^F4/80^+^ TAM subsets of 4T1 tumors were subjected to flow cytometry to measure CD86 expression. (**E**) The mRNA levels of *Il10* were measured in TAMs. *, ** Significantly different between groups compared (* *p* < 0.05; ** *p* < 0.01).

The stress-responsive enzyme, HO-1 is often upregulated in tumor tissues, and its expression is further increased in response to therapies [29]. We first examined the prognostic significance of the expression level of HO-1 using the public dataset. Kaplan-Meier analysis revealed the correlation between expression levels of HO-1 and prognosis of breast cancer. The probabilities of relapse free survival (RFS) among ER-PR-HER2- breast cancer patients who received chemotherapy were significantly reduced with an increased *Hmox-1* transcript level (Figure 4A). However, these predictions are just based on the association, and do not necessarily reflect the cause-result relationship. HO-1 is a stress responsive enzyme, and its gene expression is prone to be inducible by various external and internal factors.

HO-1 is highly expressed in TAMs in TME [24]. We first attempted to confirm its gene expression in macrophages in the 4T1 murine breast cancer model. *Hmox1* mRNA expression levels of tumor-infiltrating myeloid cells were enhanced in the PTX-treated 4T1 breast cancer group relative to an untreated 4T1 breast cancer group (Figure 4B). Consistent with the mRNA levels, the protein expression of HO-1 in TAMs was also increased in the PTX treated group compared to the untreated group (Figure 4C). To determine whether HO-1 upregulation contributes to the M2 polarization of macrophages we used ZnPP, which is a chemical inhibitor of HO-1. As shown in Figure 4D, the expression of an M1 macrophage marker, CD86 was upregulated in mice injected with PTX plus ZnPP compared to that in mice treated with PTX alone. In contrast, the PTX-induced upregulation of the M2 marker gene, *Il10* in CD11b^+^ myeloid cells isolated from 4T1 tumor-bearing mice was abolished by administration of ZnPP (Figure 4E). These results suggest that inactivation of HO-1 stimulates the polarization of TAMs toward the M1 phenotype during chemotherapy.

**Figure 5:**
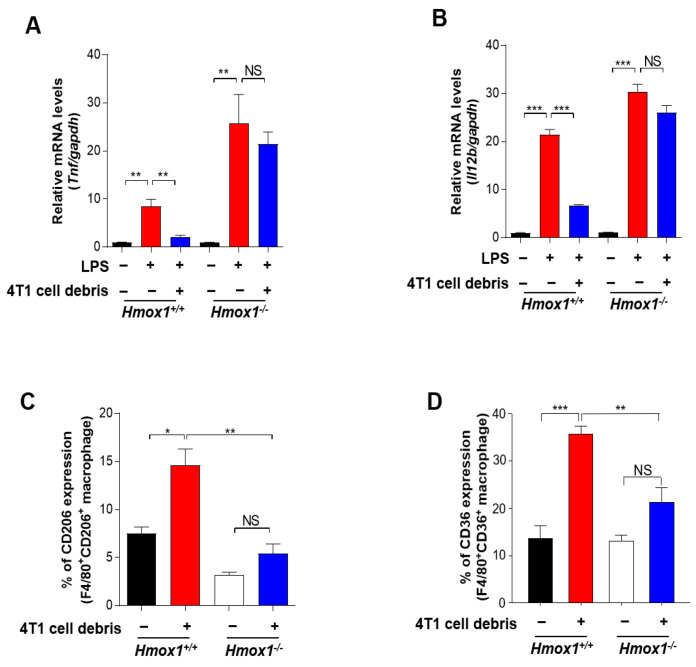
Modulation of macrophage polarization by tumor cell debris-induced HO-1. (**A**,**B**) BMDMs derived from WT or HO-1 KO mice were treated with LPS (100 ng/mL) for 24 h. The mRNA expression levels of *Tnf* (**A**) and *Il12b* (**B**) in the above-treated macrophages co-cultured with or without 4T1 cell debris for 30 h were measured by qPCR. (**C**,**D**) BMDMs from WT or HO-1 KO mice were co-cultured with or without 4T1 tumor cell debris. After 24 h of incubation, the proportion of macrophages expressing CD206 (**C**) or CD36 (**D**) was assessed by flow cytometry. *, **, *** Significantly different between groups compared (* *p* < 0.05; ** *p* < 0.01; *** *p* < 0.001). NS; not significant.

Notably, the genetic ablation of HO-1 abolished the inhibitory effect of 4T1 tumor cell debris on expression of two representative M1 marker genes, *Tnf* (Figure 5A) and *Il12b* (Figure 5B), in LPS-stimulated BMDMs. We then investigated the possible involvement of HO-1 in M2 polarization. IL-10 is known to play a role in M2-like polarization in murine macrophages. The inhibition of HO-1 activity with ZnPP decreased the expression of *Il10* (Figure S2B) and the proportion of BMDMs expressing CD206 (Figure S2C). HO-1-deficient BMDMs exposed to tumor cell debris also exhibited a diminished expression of CD206 (Figure 5C) and the scavenger receptor CD36 (Figure 5D) compared to the corresponding wild type (WT) macrophages. Likewise, both constitutive and tumor cell debris-induced expression of CD36 was downregulated in the ZnPP-treated macrophages (Figure S2F). Taken together, these results suggest that HO-1 signaling induced by tumor cell debris modulates the polarization of macrophages into an M2 phenotype in the TME following chemotherapy. We found that BMDMs from HO-1 knock out (KO) mice did not significantly differ from WT macrophages in their ability to take up tumor cell debris (Figure S3A,B). Therefore, anti-tumor strategies could potentially target the HO-1-mediated reprogramming of TAMs without altering their clearance efficiency.

### 3.4. HO-1 Inhibition Potentiates Anti-Tumor T Cell Function in Response to PTX Treatment

**Figure 6:**
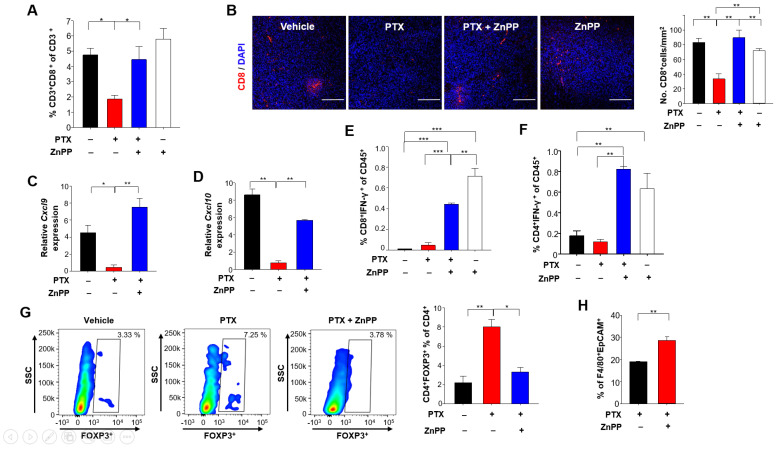
Effects of HO-1 inhibition on T cell-mediated anti-tumor immunity in 4T1-tumor bearing mice under PTX therapy. (**A**) CD45^+^CD3^+^CD8^+^ tumor-infiltrating lymphocyte populations (as percentages) were identified by flow cytometry. (**B**) Representative images of tumor sections stained for CD8^+^ T cells and DAPI are shown. Scale bar, 200 μm. (**C**,**D**) The whole-tumor mRNA expression levels of *Cxcl9* (**C**) and *Cxcl10* (**D**) were analyzed by qPCR. (**E**) The percentages of IFN-γ^+^CD8^+^ T cells in CD45^+^ populations were detected by flow cytometry. (**F**) The proportions of IFN-γ^+^CD4^+^ T cells in the CD45^+^ populations were analyzed by flow cytometry. (**G**) The percentages of Foxp3^+^CD4^+^ T cells were analyzed by flow cytometry. (**H**) Engulfment of breast tumor cell debris was analyzed as the proportion of F4/80^+^ macrophages found to contain intracellular EpCAM (a standard epithelial tumor cell marker), as assessed by flow cytometry. *, **, *** Significantly different between groups compared (* *p* < 0.05; ** *p* < 0.01; *** *p* < 0.001).

In the context of an anti-tumor immune response, a sufficient number of infiltrating T cells is needed to achieve a favorable clinical outcome. Notably, there were significantly more CD8^+^ T cells in tumors of mice treated with PTX plus ZnPP compared to those from mice treated with PTX alone (Figure 6A,B). Elevated levels of the chemokines, such as CXC-chemokine ligand 9 (CXCL9) and CXC-chemokine ligand 10 (CXCL10) are reportedly associated with the increased number of tumor-infiltrating CD8^+^ T cells [30]. Therefore, we examined whether HO-1 inhibition could accelerate the infiltration of CD8^+^ T cells by upregulating expression of these chemokines. Indeed, the mRNA levels of *Cxcl9* (Figure 6C) and *Cxcl10* (Figure 6D) were higher in tumors from mice treated with PTX plus ZnPP, and this coincided with enhanced CD8^+^ T cell recruitment.

The functions of cytotoxic T lymphocyte effectors are mediated through secretion of cytokines, such as interferon (IFN)-γ [31]. The proportion of T cells producing IFN-γ was significantly increased in mice treated with PTX and ZnPP compared to those treated with PTX alone (Figure 6E,F). Furthermore, the proportion of FOXP3^+^ regulatory T cells (Tregs) from 4T1 tumor-bearing mice injected with PTX plus ZnPP was lower than that from 4T1 tumor-bearing mice treated with PTX alone (Figure 6G). Consistent with the ability of PTX plus ZnPP to increase the anti-tumor immune responses, the same combined treatment increased the proportion of macrophages phagocytosing breast tumor cell debris to a greater extent than that achieved with the chemotherapy alone (Figure 6H).

### 3.5. HO-1 Inactivation-induced M1 TAMs Are Crucial for the Enhanced Response to PTX Therapy

**Figure 7:**
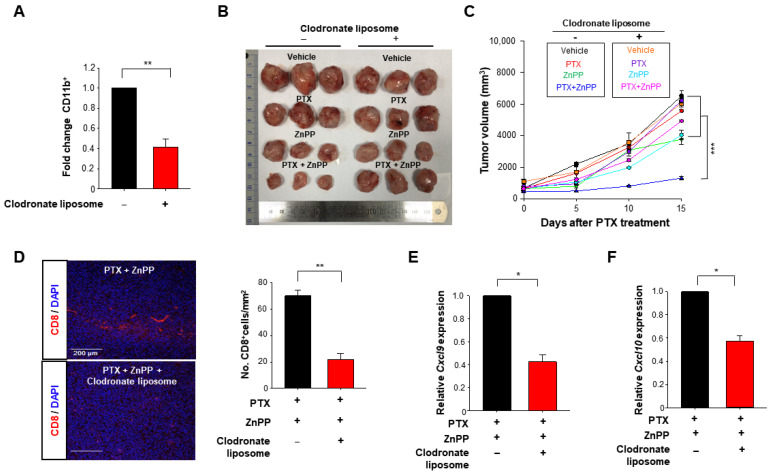
Attenuation of anti-tumor activity of PTX plus an HO-1 inhibitor by macrophage depletion in a murine syngeneic breast tumor model. (**A**) Macrophage depletion was confirmed by flow cytometry. (**B**) 4T1 breast cancer-bearing mice were injected with vehicle or PTX (5 mg/kg, 5 days) in combination with daily intraperitoneal injection of ZnPP (40 mg/kg). For macrophage depletion, mice were treated with clodronate liposomes 3 times at 4-day intervals as described in Materials and Methods. Representative photographs of tumors are shown for each group. (**C**) Tumor volume was calculated as a relative change and plotted as the mean ± SEM. (**D**) Representative images of tumor sections stained for CD8^+^ T cells and DAPI are shown. (**E**,**F**) The whole-tumor mRNA expression levels of *Cxcl9* and *Cxcl10* were analyzed by qPCR. *, **, *** Significantly different between groups compared (* *p* < 0.05; ** *p* < 0.01, *** *p* < 0.001).

To further determine whether macrophages are necessary to support the T cell function in PTX and ZnPP co-treatment, we used a clodronate liposome to deplete macrophages (Figure 7A). We then compared the effects of macrophage depletion on therapeutic activity of PTX and ZnPP in the 4T1 syngeneic murine breast tumor model (Figure S3C). Of note, the tumor-inhibiting efficacy of PTX and ZnPP co-treatment was abrogated in the macrophage depletion group (Figure 7B,C). Macrophage depletion also reduced the intratumoral CD8^+^ T cell population (Figure 7D) and attenuated the expression of M1-associated genes, *Cxcl9* (Figure 7E) and *Cxcl10* (Figure 7F) in mice treated with PTX and ZnPP.

## 4. Discussion

HO-1 in TAMs modulates immunosuppressive TME [29,32,33]. Here, we found that inhibition of tumor cell debris-induced HO-1 expression in TAMs potentiates anti-tumor functions of T cells. Tumor cell debris formed as a consequence of chemotherapy is a key factor to reprogram the macrophages in the breast cancer. The macrophage polarization is subjected to redox regulation [34]. HO-1 expression is mainly regulated by the redox sensitive transcription factor Nrf2 that plays vital roles in cellular protection against oxidative stress and inflammatory insults [24]. After the engulfment of cell debris, the macrophages form phagolysosome to degrade the cell debris. This results in the high acidity condition in macrophages and generation of reactive oxygen species (ROS) [35]. The tripeptide glutathione (GSH) eliminates/neutralizes ROS and consequently protects cells from oxidative stress [36]. The GSH levels determine the intracellular redox status and hence influence macrophage polarization [36]. Collectively, tumor cell debris-induced macrophage reprogramming could be regulated by intracellular redox status.

The major approaches currently used to target TAMs for anti-cancer therapy include inactivating/depleting them or reprogramming to attain an anti-tumor phenotype [37,38]. The targeting of PS receptors, which recognize dead tumor cells, has been shown to yield therapeutic effects by reprogramming TAMs to the M1 phenotype [39,40,41]. However, blocking PS receptors present on the surface of macrophages could decrease their clearance of dead tumor cells [40], which may influence the therapeutic efficacy of anticancer treatment [42]. It has been suggested that it may be unwise to deplete TAMs in combination with cytotoxic anti-tumor therapy due to the potential for aberrant immune responses [43,44]. Thus, it is far more attractive to consider targeting macrophages for anti-cancer therapy while enabling them to maintain ability to efficiently clear dead cancer cells [43]. We found that HO-1-deficient macrophages did not significantly differ from WT macrophages in their ability to take up tumor cell debris (Figure S2B,C). Therefore, anti-tumor strategies could potentially target the HO-1-mediated reprogramming of TAMs without altering their clearance efficiency.

Previous studies have shown that therapeutic inefficacy can be caused by Tregs, which suppress lymphocytic activity via HO-1-mediated mechanisms [45,46]. M2 TAMs play a key role in enhancing the immunosuppressive activity of Tregs through IL-10 [36]. In line with the decreased mRNA levels of *Il10* in TAMs observed herein, we found that the proportion of Tregs was decreased in mice treated with PTX and a HO-1 inhibitor compared to that in mice treated with PTX alone. Modulation of the ability of conventional chemotherapy-generated tumor cell debris to change the TME could be a novel approach for preventing unwanted therapy-induced effects [21,22]. Recent studies have shown that 5-FU-generated tumor cell debris stimulates tumor growth [22]. Although we focused on PTX in the present study, 5-FU, which is another first-line chemotherapeutic agent, also showed similar results in a 4T1 breast cancer model (S.H. Kim and Y.-J, Surh, unpublished observation).

The fact that dying/dead cancer cells may orchestrate tumor repopulation warns about the dark side of cytotoxic therapy, so we need to develop new strategies to overcome this paradoxical dilema [23]. The low-dose metronomic (LDM) chemotherapy is a form of continuous use of low doses of conventional chemotherapeutics [47]. This strategy has been utilized in elderly patients with lower dose treatment than conventional maximum tolerated dose chemotherapy [47,48]. In particular, LDM is an emerging therapeutic strategy for treating metastatic breast cancer though its mechanistic basis remains to be clarified [48]. The maximal dose of conventional chemotherapy generates tumor cell debris which can accelerate the immunosuppressive M2-like macrophage polarization in TME. On the other hand, the LDM sustains therapeutic efficacy by generating relatively small amounts of tumor cell debris, so can be clinically beneficial as an emerging alternative to conventional chemotherapy [48].

In summary, the inactivation of HO-1 in tumor cell debris-phagocytosing macrophages critically polarizes them toward the M1 phenotype, which contributes to enhancement of cytotoxic T cell responses. Therefore, the HO-1-targeted reprogramming of TAMs toward the M1 phenotype may be a promising strategy for maximizing the efficacy of chemotherapy.

## Figures and Tables

**Table 1:** The sequences of primers used for PCR amplification.

Gene Symbol	Primer Sequences
*Hmox1*	Forward: GATAGAGCGCAACAAGCAGAA
	Reverse: CAGTGAGGCCCATACCAGAAG
*Cxcl9*	Forward: GGAGTTCGAGGAACCCTAGTG
	Reverse: GGGATTTGTAGTGGATCGTGC
*Cxcl10*	Forward: CCAAGTGCTGCCGTCATTTTC
	Reverse: GGCTCGCAGGGATGATTTCAA
*Il10*	Forward: GCTCTTACTGACTGGCATGAG
	Reverse: CGCAGCTCTAGGAGCATGTG
*Tnf*	Forward: CCCTCACACTCAGATCATCTTCT
	Reverse: GCTACGACGTGGGCTACAG
*Il12b*	Forward: TGGTTTGCCATCGTTTTGCTG
	Reverse: ACAGGTGAGGTTCACTGTTTG
*Actb*	Forward: TGCTAGGAGCCAGAGCAGTA
	Reverse: AGTGTGACGTTGACATCCGT
*Gapdh*	Forward: GGGAAGCCCATCACCATCT
	Reverse: CGGCCTCACCCCATTTG

## Data Availability

Data presented in this study are included in the article and its supplementary material file.

## Supplementary Materials

The following are available online at https://www.mdpi.com/2076-3921/10/3/470/s1. Figure S1: Effect of PTX on 4T1 tumor growth in mice. Figure S2: Modulation of macrophage polarization by tumor cell debris-induced HO-1. Figure S3: Comparison of ability of WT or HO-1 KO macrophages to clear tumor cell debris.
