# Supplementary material for: Reprograming of Tumor-Associated Macrophages in Breast Tumor-Bearing Mice under Chemotherapy by Targeting Heme Oxygenase-1
Source: Antioxidants (Basel). 2021 Mar 16;10(3):470. doi: 10.3390/antiox10030470 (PMC8002331; doi:10.3390/antiox10030470)
Supplement: Supplementary file 1 [file antioxidants-10-00470-s001.pdf]

antioxidants

MDPI

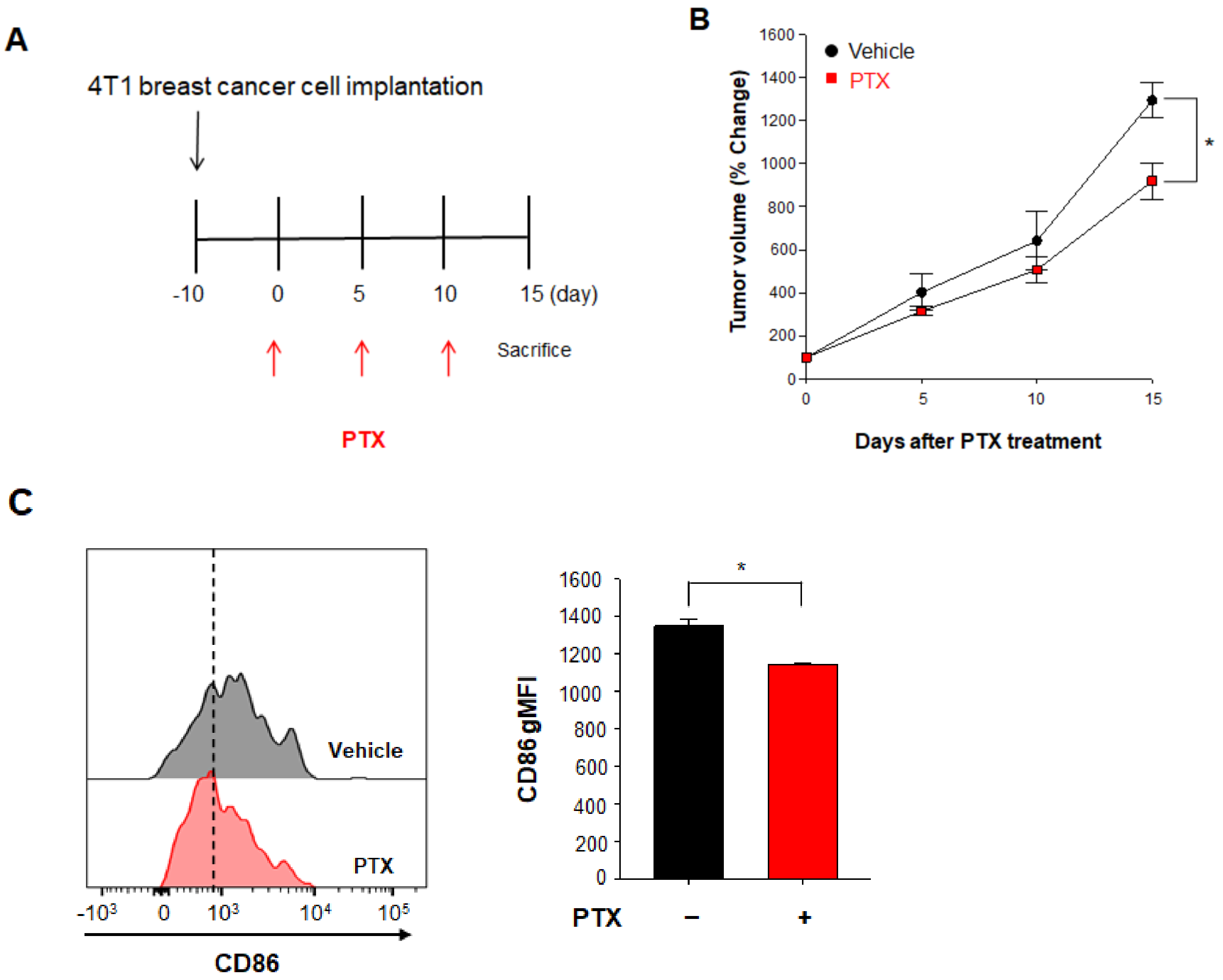

Figure S1. Effect of PTX on 4T1 tumor growth in mice. (A) Mice were implanted subcutaneously in the mammary gland with 4T1 tumor cells. At 10 days after tumor cell injection, the mice were subjected to intraperitoneal injection of vehicle or PTX (5 mg/kg) every 5 days. Mice were sacrificed on day 15 after PTX treatment and tumors were collected. (B) Effect of PTX on growth of implanted 4T1 murine mammary adenocarcinoma. The relative volume change is plotted as the total mean ± SEM. (C) The CD45$^+$CD11b$^+$Ly6G$^-$Ly6C$^-$F4/80$^+$ TAM subsets of 4T1 tumors were gated and analyzed for CD86 expression by flow cytometry. The mean fluorescence intensity (MFI) of CD86$^+$ TAMs is shown for each group. *Significantly different between groups compared ($P < 0.05$).

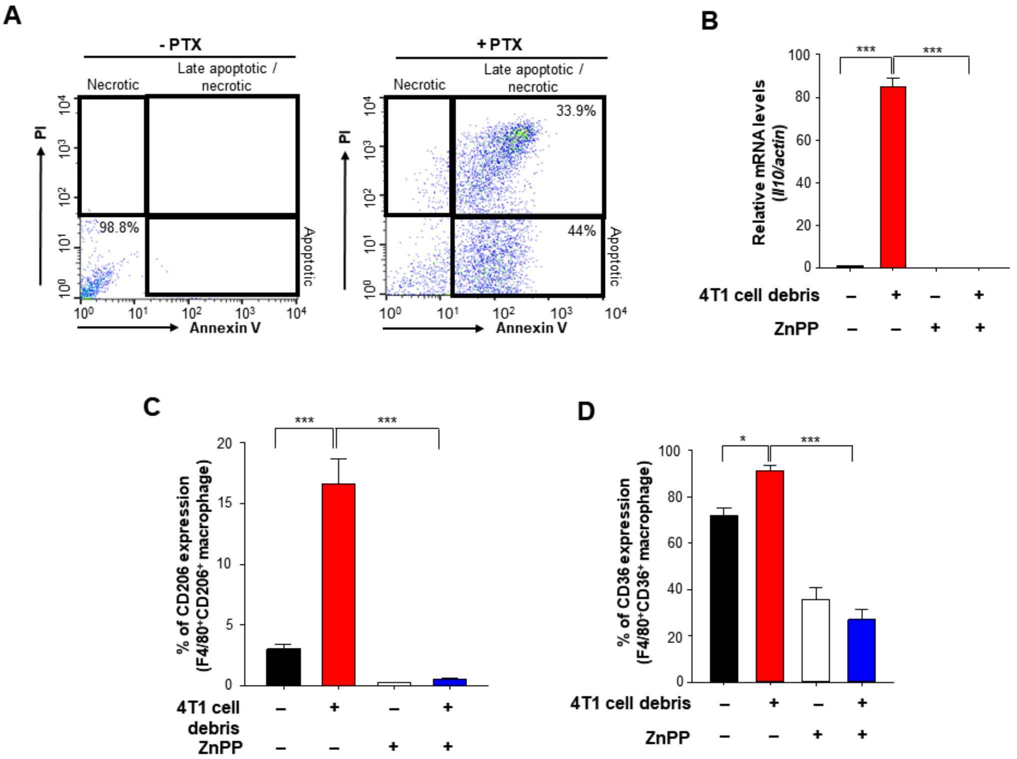

Figure S2. Modulation of macrophage polarization by tumor cell debris-induced HO-1. (A) Characterization of chemotherapy-generated tumor cell debris by annexin V/PI flow cytometry analysis. The analysis of apoptotic, necrotic and late apoptotic/necrotic tumor cell debris from 4T1 cells treated with PTX for 24 h was conducted by flow cytometry. (B) WT BMDMs pre-treated with ZnPP (10 μM) for 1 h were co-incubated with or without 4T1 breast cancer cell debris for 8 h, and the mRNA levels of *Il10* were analyzed by qPCR. (E) WT BMDMs pre-treated with ZnPP (10 μM) for 1 h were co-cultured with or without 4T1 tumor cell debris. After 24 h of incubation, the CD206 expression of macrophages was assessed by flow cytometry. (C) Macrophages pre-treated with ZnPP (10 μM) for 1 h were co-incubated with or without breast cancer cell debris for 24 h, and the expression of CD36 in macrophages was assessed by flow cytometry.

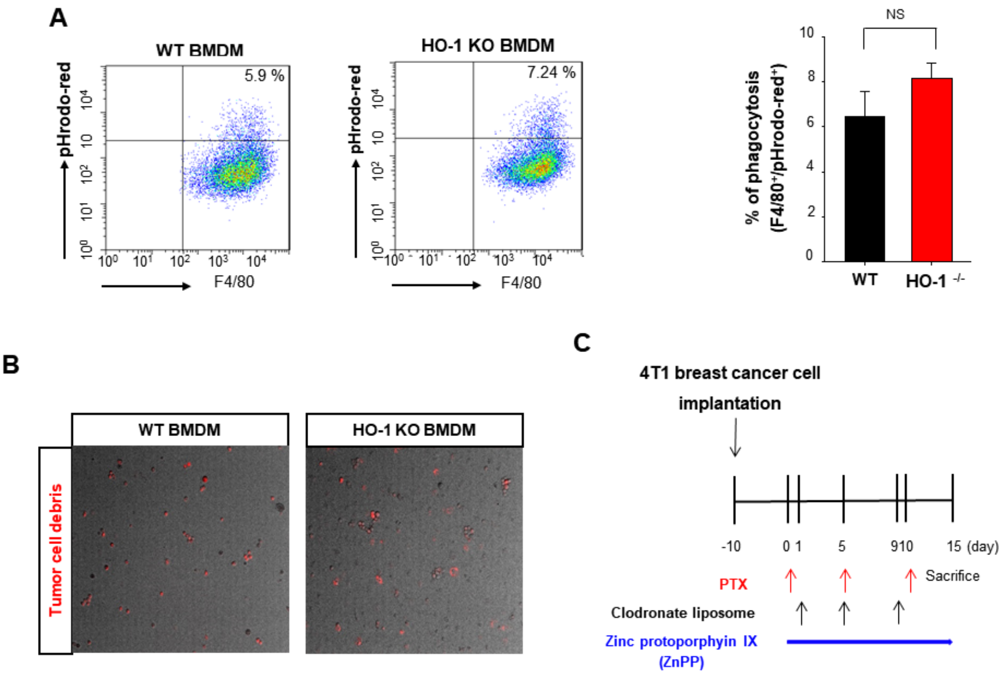

Figure S3. Comparison of ability of WT or HO-1 KO macrophages to clear tumor cell debris. (A, B) Macrophages from WT or HO-1 KO mice were co-incubated with tumor cell debris. The phagocytosis of cell debris was quantified as the proportion of macrophages (F4/80$^+$) containing intracellular tumor cell debris (pHrodo$^+$), as assessed by flow cytometry (A) and immunofluorescence (B). Data were analyzed by the Student's *t*-test. NS: not significant. (C) 4T1 breast cancer-bearing mice were injected with vehicle or PTX (5 mg/kg) 3 times at 5-day intervals in combination with daily intraperitoneal injection of ZnPP (40 mg/kg). For macrophage depletion, mice were treated with clodronate liposomes (1 mg/mouse) on day 1, 5, and 9. NS; not significant.
